# LASSBio-1135: A Dual TRPV1 Antagonist and Anti-TNF-Alpha Compound Orally Effective in Models of Inflammatory and Neuropathic Pain

**DOI:** 10.1371/journal.pone.0099510

**Published:** 2014-06-18

**Authors:** Cleverton K. F. Lima, Rafael M. Silva, Renata B. Lacerda, Bruna L. R. Santos, Rafaela V. Silva, Luciana S. Amaral, Luís E. M. Quintas, Carlos A. M. Fraga, Eliezer J. Barreiro, Marília Z. P. Guimaraes, Ana L. P. Miranda

**Affiliations:** 1 Laboratório de Estudos em Farmacologia Experimental (LEFEx), Faculdade de Farmácia, Universidade Federal do Rio de Janeiro, Rio de Janeiro, RJ, Brazil; 2 Instituto de Ciências Biomédicas, Universidade Federal do Rio de Janeiro, Rio de Janeiro, RJ, Brazil; 3 Laboratório de Avaliação e Síntese de Substâncias Bioativas (LASSBio), Faculdade de Farmácia, Universidade Federal do Rio de Janeiro, Rio de Janeiro, RJ, Brazil; Boston Children’s Hospital and Harvard Medical School, United States of America

## Abstract

LASSBio-1135 is an imidazo[1,2-a]pyridine derivative with high efficacy in screening models of nociception and inflammation, presumed as a weak COX-2 inhibitor. In order to tease out its mechanism of action, we investigated others possible target for LASSBio-1135, such as TNF-α and TRPV1, to better characterize it as a multitarget compound useful in the treatment of chronic pain. TRPV1 modulation was assessed in TRPV1-expressing Xenopus oocytes against capsaicin and low pH-induced current. Modulation of TNF-α production was evaluated in culture of macrophages stimulated with LPS. *In vivo* efficacy of LASSBio-1135 was investigated in carrageenan and partial sciatic ligation-induced thermal hyperalgesia and mechanical allodynia. Corroborating its previous demonstration of efficacy in a model of capsaicin-induced hyperalgesia, LASSBio-1135 blocks capsaicin-elicited currents in a non-competitive way with an IC_50_ of 580 nM as well as low pH-induced current at 50 µM. As an additional action, LASSBio-1135 inhibited TNF-α release in these cells stimulated by LPS with an IC_50_ of 546 nM by reducing p38 MAPK phosphorilation. Oral administration of 100 µmol.Kg^−1^ LASSBio-1135 markedly reduced thermal hyperalgesia induced by carrageenan, however at 10 µmol.Kg^−1^ only a partial reduction was observed at the 4^th^ h. Neutrophil recruitment and TNF-α production after carrageenan stimulus was also inhibited by the treatment with LASSBio-1135. Modulating TRPV1 and TNF-α production, two key therapeutic targets of neuropathic pain, 100 µmol.Kg^−1^ LASSBio-1135 was orally efficacious in reversing thermal hyperalgesia and mechanical allodynia produced by partial sciatic ligation 7–11 days after surgery without provoking hyperthermia, a common side effect of TRPV1 antagonists. In conclusion LASSBio-1135, besides being a weak COX-2 inhibitor, is a non-competitive TRPV1 antagonist and a TNF-α inhibitor. As a multitarget compound, LASSBio-1135 is orally efficacious in a model of neuropathic pain without presenting hyperthermia.

## Introduction

The pharmacological treatment of patients with chronic pain is a current challenge, since existing drugs have little efficacy and present serious side effects. The two major types of chronic pain, inflammatory and neuropathic pain, are mostly treated with drugs that alleviate the symptoms without affecting the underlying disease, such as nonsteroidal and steroidal anti-inflammatory drugs, opioids, antidepressants, and anticonvulsants, depending on the nature of pain. Controlling the inflammatory response is essential not only in inflammatory chronic pain, as pointed by recent studies that have shown the role of inflammation in the development of neuropathic pain and consequently have supported new therapeutic approaches that target immune response [1].

LASSBio-1135 belongs to a series of imidazo[1,2-*a*]pyridine derivatives designed as dual inhibitors of p38 mitogen-activated protein kinase (p38 MAPK) and cyclooxigenase-2 (COX-2) based on the structures of SB203580 (p38 MAPK inhibitor) and celecoxib (COX-2 selective inhibitor) [2]. The *in vivo* pharmacological screening for anti-inflammatory and antinociceptive activities pointed out this compound as one of the most prominent of the series, since LASSBio-1135 reduced the carrageenan-induced paw edema and it completely abrogated capsaicin-induced thermal hyperalgesia. However, *in vitro* studies regarding its mechanism of action derivative showed that it did not inhibit p38 MAPK activity as planned, but it inhibited weakly COX-2 activity, reducing TXB_2_ production in whole blood stimulated with lipopolysaccharide (LPS) (IC_50_ = 18,5 µM) [2]. Therefore, the robust *in vivo* actions of this compound were not consistent with the meager *in vitro* activities, suggesting that other mechanisms might be involved in these actions.

LASSBio-1135 high efficacy in capsaicin-induced hyperalgesia could indicate the transient receptor potential vanilloid receptor type 1(TRPV1) as a possible target. TRPV1 is a nonselective cation channel expressed in subtypes of nociceptive neurons that is activated by physical or chemical stimuli, including capsaicin (CAP), heat, protons and endogenous cannabinoids (CB) [3]; [4]. Recent studies have described that TRPV1 activation is essential for the establishment of inflammation and pain in models of arthritis, showing that the expression of this receptor is increased, contributing to enhanced thermal sensitivity [5]; [6]; [7]. In addition to its effects on inflammatory pain, TRPV1 also contributes to neuropathic pain, as its expression is up-regulated in uninjured and down-regulated in injured fibers after partial nerve injury, and its blockade reduces pain sensitivity in nerve injury models [8]; [9]. Interestingly, Chen *et al*. [10] showed recently that TRPV1 plays a role in spinal cord glial activation after partial nerve injury and consequently this channel may also modulate glial activation during neuropathic pain and arthritis. Thus, several TRPV1 antagonists have been developed as a useful pharmacological alternative to treat chronic pain, some of them reaching clinical trials, however most studies were discontinued due to the main side effect presented in humans: hyperthermia [11].

The inflammatory mediators produced by immune cells, such as cytokines and chemokines, play essential roles in the development of inflammatory and neuropathic pain, acting both in the peripheral and in the central nervous system (CNS). Among the cytokines, it is well known that tumor necrosis factor alpha (TNF-α) is released by immune, immune-like glial and glial cells, in the context of inflammatory and neuropathic pain [12]. TNF-α emerges as another target for LASSBio-1135 action, since it was designed as a p38 MAPK inhibitor. As mentioned previously, LASSBio-1135 does not inhibit directly p38 MAPK activity as predicted, however its structure has the imidazo-pyridine moiety present in several inhibitors of the signaling pathways involved in TNF-α production [13], which supports the idea of TNF-α modulation. Several lines of evidence have demonstrated that TNF-α contributes to the degeneration of the injured nerve and to sensitize the primary afferents neurons directly, or indirectly through the induction of other mediators [14]; [15]; [16]. In the dorsal root ganglion (DRG), TNF-α induces the macrophage recruitment after adjuvant-induced arthritis and nerve injury. It also modulates TTX-resistant Na^+^ channels, by mechanisms that are p38 MAPK activation-dependent, and sensitizes TRPV1 [17]; [18]; [19]; [20]. Furthermore, it has been observed that TNF-α produced by glial cells in the dorsal horn of the spinal cord may also sensitize these neurons, changing their sensitivity [21]. Hence, anti-TNF-α therapies have successfully been evaluated in animal models of neuropathic and inflammatory pain [22].

Here, we show that LASSBio-1135 acts as a multitarget compound, inhibiting TNF-α production both *in vitro* and *in vivo*, and inhibiting TRPV1 currents *in vitro*, actions that could explain the diminished inflammatory and nociceptive responses in models of inflammatory and neuropathic pain. In addition, these LASSBio-1135 antinociceptive effects were present in doses in which the animals did not develop hyperthermia, the main side effect of classical TRPV1 antagonists.

## Methods

All procedures involving animals were carried out under the approval by the Comissão de Ética no Uso de Animais from the Federal University of Rio de Janeiro (CEUA-UFRJ; FARMACIA04) and were in accordance with international guidelines. In experiments with animals, mice and rats of both sexes from the Faculty of Pharmacy breeding unity were used and at the end of the experiments animals were euthanized in CO_2_ chamber.

### Materials

The rat TRPV1 clone was a generous gift from Dr. David Julius. Drugs used in this study were purchased from the following sources: capsaicin (Alexis or Sigma), dimethylsulfoxide (DMSO) (Sigma or Vetec), arabic gum (Sigma), carrageenan (Cialgas), ketamine (Syntec), xylazine (König), RPMI 1640 (Sigma), Lipopolysaccharide (LPS) from *E. coli* (Sigma), Thioglycollate (Sigma), foetal bovine serum (FBS) (Gibco), ELISA Kit for TNF-α (BD Bioscience). LASSBio-1135 was synthesized in our laboratory as described before [2].

### Oocyte Preparation and Electrophysiology

Oocytes expressing TRPV1 were obtained as described previously [23]. Briefly, adult *Xenopus laevis* female frogs were anaesthetized with tricaine and part of the ovary was surgically removed. The removed tissues was placed in a saline solution containing (in mM) 96 NaCl, 2 KCl, 5 MgCl_2_, 5 HEPES at pH 7.6, and were then treated with collagenase (Type 1, 0.8 mg.ml^–1^, Worthington) to remove the follicular membrane. Oocytes were injected with approximately 2.0 ng of rat TRPV1 cRNA obtained with mMESSAGE mMACHINE T7 (Ambion), by using a nanoliter injector. Oocytes were maintained in ND-96 (in mM: 96 NaCl, 2 KCl, 1.8 CaCl_2_, 1 MgCl_2_, 5 HEPES) supplemented with 40 µg.ml^–1^ gentamicin for 5–7 days before analysis. For recording, oocytes were placed in a small chamber under continuous superfusion with ND-96 (without gentamicin), at a flow rate of approximately 1 ml.min^–1^, and the same solution was used to dilute the test compounds, except when acidic pH was applied. In the latter case, the solution used was composed of (in mM): 96 NaCl, 2 KCl, 1 MgCl_2_, 0.1 CaCl_2_ and 5 sodium acetate, pH 5.5. Two electrode voltage-clamp recordings were obtained at room temperature (20–22°C) under a holding potential of −60 mV, using Geneclamp 500 amplifier (Axon Instruments). Electrodes were obtained on a horizontal puller (P-97, Sutter) aiming to achieve a final resistance of 0.6–1.2 MΩ and were filled with 3 M KCl. Recordings were digitized with a MacLab A/D converter (AD Instruments) at 100 Hz and digitally filtered at 2 Hz (low pass). Drug stock solutions were made in ethanol (capsaicin) or DMSO (LASSBio-1135) and were diluted in ND-96 just before the experiments, in a way that final ethanol and DMSO concentrations did not exceed 0.1% and 0.2%, respectively, and appropriate controls were tested. The solutions were exchanged via a programmable solenoid pinch valve controller (AutoMate Scientific Inc.) and were generally applied in 45-second pulses. Each pulse of LASSBio-1135 in admixture with other agents was immediately preceded by LASSBio-1135 alone in the same concentration, to allow drug equilibration. As a control, we performed an experiment with 3 consecutive applications of CAP.

### LPS-induced TNF-α Production in Cultures of Mice Peritoneal Macrophages

BALBc mice were stimulated with 3% thyoglicollate (1 ml per mouse; i.p.) and 3 days later the peritoneal cavity was washed with RPMI 1640 and the peritoneal macrophages were plated onto 96-wells plates (30,000 cells per well) and allowed to adhere for 1 hour at 37°C in a humidified 5% CO_2_ atmosphere [30]. Subsequently, the wells were rinsed once with sterile phosphate buffer saline (PBS) and then it was added RPMI 1640 supplemented with 10% foetal bovine serum, streptomycin and penicillin (50 U.ml^−1^). Then macrophages were incubated with the vehicle or compounds for 1 hour, followed by stimulation with LPS (100 ng.ml^−1^) for 24 hours [27], after which the supernatants were collected to measure TNF-α production by ELISA.

### Cell Viability: MTT Assay

Cell viability was evaluated using (3-(4,5-dimethylthiazol-2-yl)-2,5-diphenyltetrazolium bromide (MTT) assay in culture of macrophages. Briefly, murine peritoneal macrophages were obtained and cultivated as described above. After 18 h of stimuli with LPS was add 20 µl of MTT solution (5 mg.ml^−1^) and allowed the cells to incubate for 4 h. At the end, it was add 200 µl of DMSO to dissolve formazan and the absorbance was read at 532 nm.

### Western Blot Analysis

Immunoblotting was performed with lysates from cultured peritoneal macrophages obtained as described above but plated on 6-wells plates (3.500.000 cells per well). After 1 h of LPS stimuli, cells were lysed with lysis buffer (1% Triton, 20 mM Tris pH 7.6, 1 mM EDTA, 150 mM NaCl, 1 mM NaF, 1 mM NaVO_3_) containing a protease inhibitors cocktail (Sigma). Aliquots of macrophages lysates containing 30 µg of proteins in each well were subjected to electrophoresis in 10% SDS-polyacrilamide gels (Bio-Rad, Miniproteam) and transferred to nitrocellulose membranes (Bio-Rad). The membranes were then blocked with 5% non-fat milk solution in TBS buffer (25 mM Tris pH 7.6, 0.2 M NaCl) and incubated overnight at 4°C with a primary antibody against phospho-p38 MAPK (1∶1000) or total p38 MAPK (1∶1000) (both from Cell Signalling). The blots were incubated for 1 h with HRP-conjugated secondary antibody (1∶5000) (Santa Cruz) at room temperature and then revealed with ECL solution (Thermo Scientific) for 2 min following exposition to Hyperfilm (Amersham Biosciences). The same blot was first immunoreacted with phospho-p38 MAPK, following by stripping with glycine at pH 2.0 and then the incubation procedure was repeated with total p38 MAPK antibody.

### Carrageenan-induced Hyperalgesia Assay

The systemic anti-hyperalgesic activity of LASSBio-1135 in a model of inflammatory pain was evaluated using carrageenan-induced thermal hyperalgesia assay. LASSBio-1135 was orally administered (100 µmol.kg^−1^ or 10 µmol.kg^−1^; 0.1 ml.20 g^−1^) as a suspension in 5% arabic gum in saline (vehicle). Control animals received an equal volume of vehicle. One hour later, the animals were injected with either 0.1 ml of 1% carrageenan solution in saline or sterile saline (NaCl 0.9%), into the subplantar surface of the right hind paws. The thermal hyperalgesia was determined using the modified hot-plate test [24]. Rats were placed individually on a hot plate with the temperature set at 51°C. The withdrawal latency response of the challenged paw was determined at 0, 30, 60, 120, 180, and 240 min post-challenge.

### Myeloperoxidase Activity

The myeloperoxidase (MPO) activity assay was performed to evaluate the leukocyte recruitment to the injured plantar tissue after carrageenan challenge [25]; [26]. The plantar tissue of the hind paw was harvested 4 h after carrageenan or saline i.pl. 0.9% injection and kept at −80°C until the day of the experiment. At the day of the experiment, samples were weighed and adjusted to 50 mgml^−1^ with 50 mM K_2_HPO_4_ buffer (pH 6.0) containing 0.5% of hexadecyl trimethylammonium bromide, following by homogenization using a Turrax (T18) for 2 min at 22,000 rpm and centrifuged at 13,000 rpm for 4 min. The supernatants were collected and aliquots of 100 µl were added to 96 well-plates together with 100 µl of development solution containing 0.167 mg.ml^−1^ of O-dianisidine dihydrochloride (OPD) and 0.015% hydrogen peroxide. The MPO activity was measured using a spectrophotometer at 450 nm (TP-reader). A standard curve of neutrophils, obtained from ratś bone marrows, was used to compare with MPO activity obtained in the samples.

### TNF-α Quantification

TNF-α quantification in injured paw was performed in skin tissues samples harvested 4 h after challenge with carrageenan or saline. Skin tissues samples were homogenised in PBS containing protease inhibitor cocktail (Sigma), centrifuged at 13.000 rpm at 4°C for 15 min, the supernatant was collected and TNF-α production was evaluated by ELISA kit (BD Biosciense).

### Neuropathic Pain Model

The anti-hyperalgesic and anti-allodynic activities of LASSBio-1135 in chronic neuropathic pain model was investigated using the partial sciatic ligation (PSL) assay described by Seltzer *et al*. [28]. Experiments were performed with adult Swiss mice of both sexes weighing from 20 to 30 g. Surgical procedures were performed under ketamine (100 mg.Kg^−1^; i.p.) and xylazine (20 mg.Kg^−1^; i.p.) anaesthesia. First, a small incision was made on the left hind leg and then the sciatic nerve ligation was made by tying a knot positioned between the last third or half of the dorsal portion of the nerve, using absorbable 4.0 silk. In sham-operated animals the nerve was exposed without ligation. The withdrawal responses were determined before surgery and after a hypernociceptive thermal stimulus caused by a radiant heat light source (Ugo Basile) positioned directly on the plantar surface of the left hind paw for thermal stimuli and using calibrated Von Frey filaments. LASSBio-1135 was administered orally at a dose of 100 µmol.Kg^−1^ once a day starting at day 5 and ending at day 13. The withdrawal latency of the left hind paw was recorded 5, 7, 9, 11 and 13 days after sciatic ligation and always one hour after the administration of LASSBio-1135 or vehicle (5% arabic gum).

### Hargreaves Test

Baseline thermal latency was evaluated using Hargreaves test. LASSBio-1135 at 100 µmol.Kg^−1^ or vehicle were orally administered and evaluation of thermal sensitivity was performed using a radiant heat light source (Ugo Basile) positioned directly on the plantar surface of the left hind paw. The withdrawal latency was evaluated 1, 2, 3, 4 and 6 h after administration of LASSBio-1135 or vehicle.

### Temperature Measurements

LASSBio-1135 was evaluated in its ability to change body temperature, using the animals submitted to PSL. Animals had their rectal temperature measured with a digital thermometer (sensitivity 0.1°C) before surgery and each day after 5 days later, when LASSBio-1135 treatment began, always 1 h after administration of the compound (100 µmol.Kg^−1^; 0.1 ml.20 g^−1^) or vehicle.

### Statistical Analyses

Peak amplitudes of digitized current traces were measured with Chart software. Results were expressed as mean ± SEM and compared with appropriate control groups. Data were statistically analyzed by the Students *t* test, One-way and Two-way ANOVA (Dunnett post-test and Bonferroni post-test) for a significance level of p<0.05. When appropriate, the IC_50_ values (*i.e*. the concentration able to inhibit 50% of the maximum effect observed) were determined by non-linear regression using GraphPad Prism software v. 5.0.

## Results

### LASSBio-1135 Inhibits Capsaicin- pH 5.5-elicited Currents in Xenopus Oocytes Expressing TRPV1

**Figure 1:**
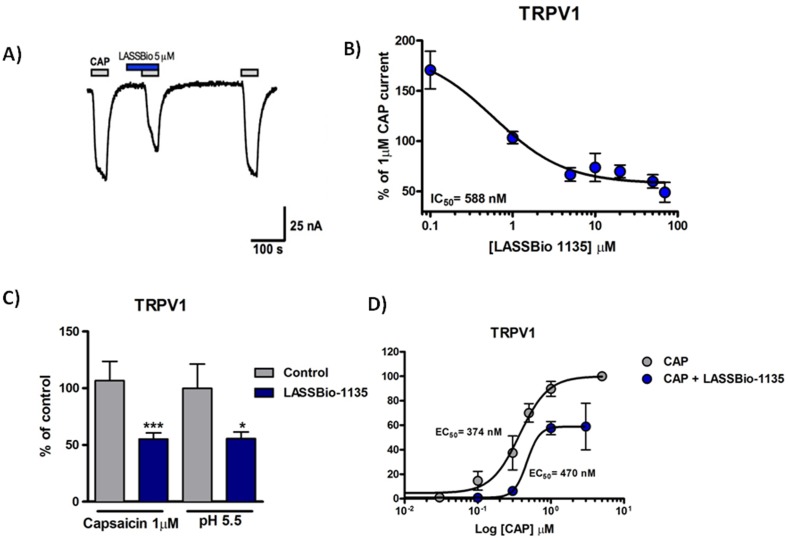
LASSBio-1135 is a non-competitive TRPV1 antagonist. A) Representative recordings of inhibition produced by LASSBio-1135 at 5 µM of 1 µM capsaicin currents in TRPV1-expressing oocyte. B) Concentration-response curve of LASSBio-1135 against 1 µM capsaicin, normalized by 10 µM capsaicin applied at the end of recording. Estimated IC_50_ was 588 nM (*n* = 5–8 oocytes per concentration). C) Quantification of peak currents in capsaicin and pH 5.5 in the presence or absence of LASSBio-1135 at 50 µM, normalized by the response to capsaicin or pH 5.5 applied at the end of recording (*n* = 6 per group). Results were analyzed using Student *t* test (*n* = 6 oocytes per group, *p<0.05 compared to control group). D) Concentration-response curves of capsaicin and capsaicin plus 5 µM of LASSBio-1135 in TRPV1 currents. Estimated EC_50_ for capsaicin were 374 nM and 470 nM, alone and in the presence of LASSBio-1135, respectively (*n* = 5–6 oocytes in each curve). IC_50_ e EC_50_ values were determined by non-linear regression using GraphPad Prism software.

As previously shown by our group in a pharmacological screening, LASSBio-1135 was orally effective in completely reducing hyperalgesia induced by capsaicin, suggesting the possibility of TRPV1 modulation [2]. To investigate whether LASSBio-1135 could modulate TRPV1 activity, *Xenopus* oocytes were injected with TRPV1 cRNA and electrophysiological recordings were performed. Firstly, oocytes were challenged with submaximal 1 µM capsaicin (CAP), then followed by CAP with co-administration of LASSBio-1135 at 5 µM. The co-application of LASSBio-1135 reduced the CAP current approximately by half which is completely reversed by final application of CAP (Figure 1A). As a control, three consecutive application of CAP do not promote desensitization phenomenon of TRPV1 in our conditions (Figure S1). LASSBio-1135 co-administration inhibited CAP-evoked currents in a concentration-dependent manner (Figure 1B, IC_50_ = 580 nM), however it is observed only a partial reversibility at higher concentrations. Next we asked whether LASSBio-1135 would also interfere with other modalities of TRPV1 activation, particularly the acidic pH, which is found in inflammatory conditions. Similarly to what observed with CAP stimulation, LASSBio-1135 at 50 µM also reduced proton-evoked currents (Figure 1C). Hence, these results suggest that this new compound might act as an antagonist of the TRPV1 receptor.

Next, to get an idea of the nature of the LASSBio-1135 antagonist effect on TRPV1, we treated oocytes with increasing concentrations of CAP with or without 5 µM LASSBio-1135. As can be observed in figure 1D, there was only a slight shift in the CAP EC_50_ in the presence of LASSBio-1135 compared with CAP alone (0.37 to 0.47 µM). However, more interestingly, in the presence of LASSBio-1135 the concentration-response curve of CAP did not reach the maximal effect (CAP = 100%; CAP+ LASSBio-1135 = 59±19%). These results suggest that LASSBio-1135 might be a non-competitive TRPV1 antagonist.

### LASSBio-1135 Inhibits TNF-α Production in LPS-stimulated Murine Peritoneal Macrophage by Reducing p38 MAPK Activation

**Figure 2:**
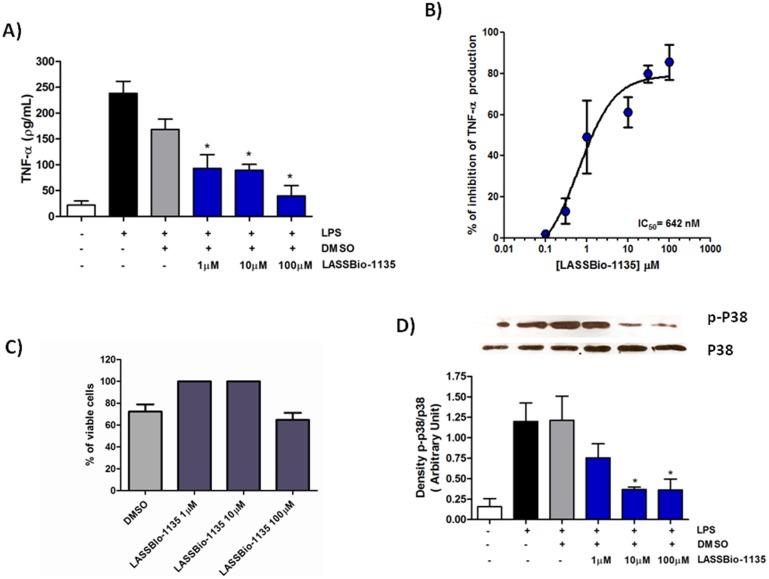
LASSBio-1135 inhibits TNF-α production in murine macrophages stimulated with LPS by blockade of p38 MAPK signaling pathway. A) Representative graph showing that LASSBio-1135 inhibits TNF-α release by macrophages stimulated with LPS (100 ng.ml^−1^) at three different concentrations. Results were analyzed using One-way ANOVA followed by Dunnett post-test (*n* = 3–4 experiments per group, *p<0.05 compared to DMSO group). B) Concentration-response curve of LASSBio-1135. The IC_50_ estimated was 642 nM (*n* = 3–4 experiments per concentration). D) LASSBio-1135 interferes with cell viability only at 100 µM. Results were analyzed using One-way ANOVA followed by Dunnett post-test (*n* = 3–4 experiments per group, *p<0.05 compared to DMSO group) C) LASSBio-1135 reduces p38 MAPK activation induced by LPS in a concentration-dependent fashion. Results were analyzed using One-way ANOVA followed by Dunnett post-test (*n* = 3 experiments per group, *p<0.05 compared to DMSO group).

LASSBio-1135 has the structural scaffold of many compounds which interfere with several proteins kinases involved in TNF-α production, raising up the possibility of immune modulation. In order to investigate this hypothesis and attribute immune modulation to LASSBio-1135 mechanism of action we evaluated whether this compound reduces TNF-α release in inflammatory cells. Murine peritoneal macrophages were stimulated with 100 ng.ml^−1^ of LPS for 24 h to induce TNF-α production. Macrophages produced a large amount of TNF-α in response to LPS which was reduced with previous incubation of increasing concentrations of LASSBio-1135 (Figure 2A), thus presenting a concentration-dependent inhibition (Figure 2B, IC_50_ = 642 nM). Moreover, LASSBio-1135 only interferes significantly with cell viability in concentrations higher than 100 µM, as shown by MTT assay (Figure 2C).

In an attempt to confirm that LASSBio-1135 could inhibit TNF-α production by interfering with signalling pathways we decided to investigate if its action could involve modulation of p38 MAPK upstream signalling pathway, since it does not inhibit p38 activity [2]. For this purpose, murine peritoneal macrophages, previously treated with different concentrations of LASSBio-1135, were stimulated with LPS and the cell lysates were submitted to immunoblotting for phosphorylated p38 [29]. LPS rapidly induced the phosphorylation of p38 MAPK compared to the unstimulated group and the cell treatment with LASSBio-1135 reduced the activation of this protein kinase in a concentration-dependent manner (Figure 2D).

### LASSBio-1135 Inhibits Thermal Hyperalgesia Induced by Carrageenan Reducing TNF-α Production and Neutrophils Recruitment

**Figure 3:**
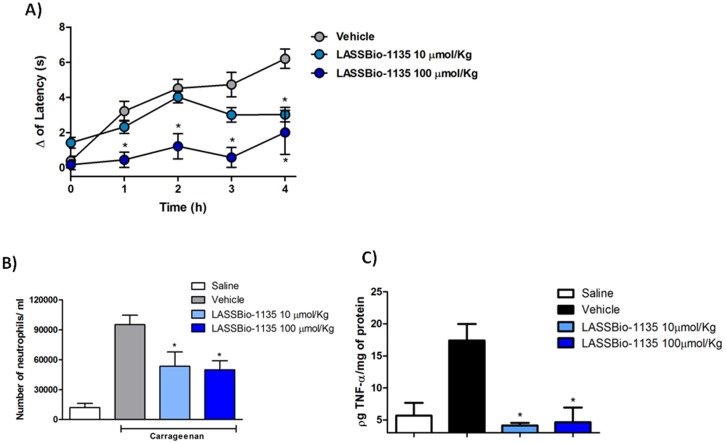
LASSBio-1135 inhibits carrageenan-induced thermal hyperalgesia by reducing TNF-α production and neutrophils infiltration in the injured paw. A) Oral pre-treatment with LASSBio-1135 at 10 and 100 µmol.Kg^−1^ 1 hour before carrageenan injection into the paw, reduces thermal hyperalgesia at different time points.Results were analyzed using Two-way ANOVA followed by Bonferroni post-test. (*n* = 6–8 animals per group, *p<0.05 compared to vehicle group). B) 4 hours after stimulus with carrageenan paws were removed and neutrophils migration was quantified by MPO activity. LASSBio-1135 treatment also reduces neutrophils migration Results were analyzed using One-way ANOVA followed by Dunnett post-test (*n* = 4 animals per group, *p<0.05 compared to vehicle group). C) The same tissue samples were used to quantify TNF-α production. LASSBio-1135 oral treatment with 10 µmol.Kg^−1^ and 100 µmol.Kg^−1^ also reduced TNF-α production in the injured paw Results were analyzed using One-way ANOVA followed by Dunnett post-test (*n* = 3–4 animals per group, *p<0.05 compared to vehicle group).

The therapeutic efficacy of LASSBio-1135 as an anti-inflammatory compound *in vivo* was proved in model of paw inflammation induced by carrageenan, however its efficacy as anti-hyperalgesic was evaluated only after capsaicin stimulation. Having demonstrated that LASSBio-1135 modulate neuronal activation by TRPV1 antagonism and immune activity by interfering with TNF-α production, we decided to investigate its efficacy to alleviate thermal hyperalgesia response in a model of inflammatory pain. Then, we used the carrageenan-induced thermal hyperalgesia assay, where we proceeded the prior administration of two doses of LASSBio-1135 (p.o.) at 100 µmol.Kg^−1^ and 10 µmol.Kg^−1^, which previously proved effective [2], followed by the i.pl. injection of carrageenan 1h later. At 100 µmol.Kg^−1^, LASSBio-1135 totally abrogated the thermal hyperalgesia since one hour after the challenge with carrageenan, while at 10 µmol.Kg^−1^ a significant reduction is observed only four hours after challenge (Figure 3A) (Baseline values: Vehicle = 12.1 s±0.4; LASSBio-1135 10 µmol.Kg^−1^ = 10.4 s±0.6; LASSBio-1135 100 µmol.Kg^−1^ = 12.9 s±0.7).

TNF-α is one of the cytokines released during carrageenan-induced inflammation and it is responsible for sensitizing primary afferent nociceptor as well as for orchestrating inflammatory response recruiting leukocytes [26]. As a TNF-α inhibitor *in vitro*, we investigated whether LASSBio-1135 could reduce hyperalgesia by hampering TNF-α production and then the recruitment of neutrophils. The injured paws were harvested and neutrophil recruitment was evaluated by the MPO-assay. Both doses of LASSBio-1135 reduced the cell recruitment in agreement with the results of hyperalgesia at the 4th h (Figures 3A and 3B). Regarding the TNF-α production, both doses of LASSBio-1135 at 100 µmol.Kg^−1^ and at 10 µmol.Kg^−1^ reduce this cytokine production in the injured paw. As observed for neutrophil recruitment, LASSBio-1135 strongly reduced TNF-α production, keeping the concentration similar to the saline treated animals (Figure 3C).

### LASSBio-1135 Reduces the Thermal Hyperalgesia and Mechanical Allodynia in Model of Neuropathic Pain

**Figure 4:**
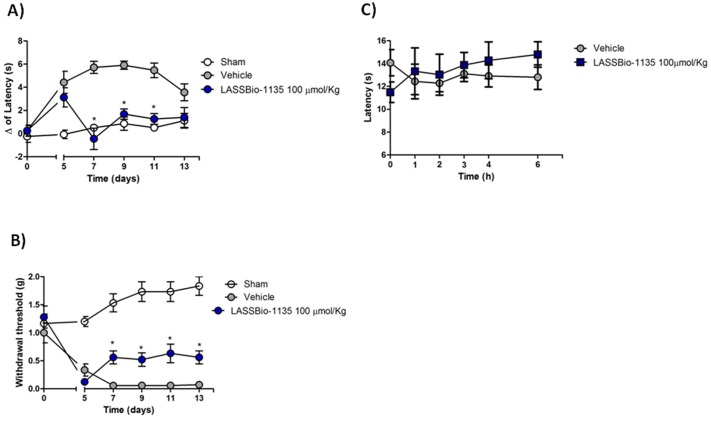
LASSBio-1135 is orally effective in reducing thermal and mechanical hypersensitivity induced by partial sciatic ligation. A) LASSBio-1135 (100 µmol.Kg^−1^; p.o.) inhibits thermal hyperalgesia induced by a radiant heat light source. Delta (Δ) of latency was obtained by comparing times of withdrawal before and after surgery. B) LASSBio-1135 (100 µmol.Kg^−1^; p.o.) also inhibits mechanical allodynia induced by Von Frey filaments. The compound was daily administered from day 5 until day 13 after surgery, 1 hour before evaluation of thermal and mechanical hypersensitivity. The withdrawal responses were determined beginning 5 days after the surgery. C) LASSBio-1135 (100 µmol.Kg^−1^; p.o.) did not affect baseline thermal sensibility induced by a radiant heat light source. Compound or vehicle were administered and evaluation of thermal sensitivity was performed at subsequent 1, 2, 3, 4 and 6 h. Results are expressed as mean ± SEM and analysed using Two-way ANOVA followed by Bonferroni post-test (*n* = 6–8 animals; *p<0.05 compared to vehicle group).

Considering the activity presented by LASSBio-1135 against TRPV1 and TNF-α, two relevant targets for the treatment of neuropathic pain based on the modification of this disease, and the efficacy presented by this compound in models of acute and inflammatory pain we decided to investigate if the oral treatment with LASSBio-1135 is effective in model of neuropathic pain induced by partial sciatic ligation. Then, 5 days after the surgery to establish nerve injury we started the daily treatment with the dose of 100 µmol.Kg^−1^ of LASSBio-1135 (p.o.), which was the most effective dose in pain assays as described above, or vehicle. The evaluation of thermal (Baseline values: Sham = 9.9 s±0.5; Vehicle = 10.2 s±0.6; LASSBio-1135 = 11.0 s±0.8) or mechanical allodynia was performed in every other day. In the first day of treatment it was not observed any change in thermal sensitivity or mechanical allodynia of injured mice compared to the vehicle group (Figure 4A and 4B). However, at the 7^th^ day post-ligation, i.e. after 3 days of treatment with LASSBio-1135, it started to be effective reducing in thermal hyperalgesia and mechanical allodynia, however it was more efficacious in reducing thermal sensitivity, where the delta of latency was similar to the sham operated mice (Δ of latency: Sham = 0.5±0.2 s; LASSBio-1135 = −0.5±0.9 s), than in reducing mechanical allodynia, where it was observer only a partial decrease in mechanical sensitivity. The anti-hyperalgesic activity of LASSBio-1135 was slightly reduced in thermal hyperalgesia and it was unaffected in mechanical allodynia, however it remained effective until the 7^th^ day of treatment (n = 6–8 animals, *p<0,05) (Figure 4A and 4B). Also, we did not observe any changes in baseline thermal sensitivity (Figure 4C).

### The Long-term Treatment with LASSBio-1135 did not Promote Hyperthermia

**Figure 5:**
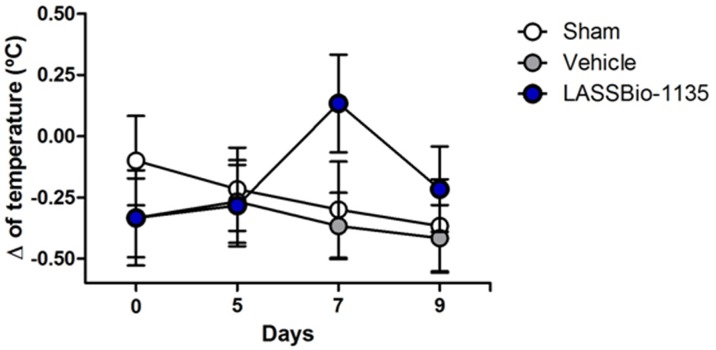
LASSBio-1135 chronic treatment did not promote hyperthermia. Animals submitted to partial sciatic ligation and daily treated with vehicle or LASSBio-1135 (100 µmol.Kg^−1^, p.o.) had their rectal temperature measured 1 hour after compound administration. Delta (Δ) of temperature was obtained comparing the measures of temperature obtained before and after surgery. Results are expressed as mean ± SEM (*n* = 6–8 animals).

Classical TRPV1 antagonists promote increase in body temperature, except those which inhibit CAP-induced currents but not proton and heat activation [30]; [31]. Thus, to prove that LASSBIo-1135 is efficacious without presenting this main side effect, we also verified the rectal temperature of mice submitted to the nerve injury and daily treated with vehicle or LASSBio-1135 (100 µmol.Kg^−1^, p.o.). Sham and vehicle treated animals did not show changes in body temperature during the days of test performance (Figure 5). Fortunately, the treatment with LASSBio-1135 did not increase significantly the body temperature of the mice, despite of promoting a slightly increase in the temperature (Δ of temperature = 0.13±0.19°C) when it starts to be effective as an anti-hyperalgesic activity (at the 7th day post-ligation). This change is not statistically significant and it is not as high as that verified for the treatment with classical TRPV1 antagonists [32].

## Discussion and Conclusions

There is growing body of evidence showing that therapeutic alternatives which control neuronal activity and immune activation might be useful in the management of chronic pain [23]; [33]; [34]. Here, we have reported that LASSBio-1135, an imidazo[1,2-*a*]pyridine derivative previously described as an analgesic and anti-inflammatory prototype inhibitor of COX-2 [2], modulates neuronal activity by antagonizing TRPV1 and immune activation by inhibiting TNF-α production. Beside this *in vitro* characterization, LASSBio-1135 was evaluated *in vivo* showing efficacy in models of acute inflammatory pain and neuropathic pain.

The search for clarifying LASSBio-1135 mechanism of action raised due to intriguingly discrepancy between its efficacy observed *in vivo* studies and its poor *in vitro* effects. The anti-hyperalgesic efficacy presented previously by oral administration of LASSBio-1135 in capsaicin-induced thermal hyperalgesia, a model where classical anti-inflammatory inhibitors of COX as celecoxib are ineffective, strongly suggested that this compound might modulate targets other than COX-2 [2], such as TRPV1. In agreement with this effect, LASSBio-1135 antagonizes TRPV1 expressed in oocytes of *X. laevis* by non-competitive mechanisms. COX inhibitory effect of LASSBio-1135 did not interfere with its action against TRPV1 observed in oocytes from *X. laevis*, since it is known that these cells express only COX-1 constitutively, which is not inhibited by LASSBio-1135 [45].

The modulation of TRPV1 channels has been suggested as a relevant target to manage painful conditions and many efforts have driven the search to find new antagonists of this receptor. TRPV1 antagonists have long been designed and successfully evaluated in models of inflammatory and neuropathic pain, being able to reduce pain-associated symptoms such as thermal hyperalgesia and even mechanical allodynia through modulation of TRPV1 in the CNS [31]. The majority were developed as competitive antagonists based on the structure of two potent TRPV1 agonists, capsaicin and resiniferatoxin (RTX), interacting with the vannilloid binding pocket antagonizing TRPV1 activation by capsaicin and protons. Meanwhile only a few compounds have been reported as non-competitive antagonists but studies with representatives of this class, such as DD161515 which presents a similar potency to LASSBio-1135, suggest the existence of a different receptor site from the CAP binding site [35]. These findings support the idea that the interaction of LASSBio-1135 with TRPV1 might not be dependent on the vanilloid binding pocket, since it blocks TRPV1 non-competitively.

In addition to neuronal activity modulation, it has been reported that the development of both inflammatory and neuropathic pain depend on the action of immune cells [36]; [17]. These cells orchestrate the inflammatory response producing chemokines, lipids mediators and cytokines, mainly the TNF-α. Recent works have pointed out the specific role of TNF-α in the development of mechanical hyperalgesia in models of arthritis [17] and in the development of neuropathic pain. The up-regulation of TNF-α production was described in the DRG of animals submitted to spinal nerve ligation [36]. Then, compounds that dampen immune cells activation, inhibiting TNF-α production could be useful to treat these two types of chronic pain. We have previously demonstrated that LASSBio-1135 reduces inflammatory response induced by carrageenan, firstly by weakly inhibiting COX-2 and now we show that this compound may also play this action by inhibiting directly TNF-α production by immune cells. TNF-α inhibitory effect is independent of TRPV1 antagonism and of COX inhibition, since macrophages and others immune cells do not express TRPV1 and many COX inhibitors, such as Celecoxib and Etoricoxib, do not interfere directly with TNF-α production in isolated macrophages (Unpublished data). In fact, as we observed a higher potency for TNF-α inhibition in macrophages (IC_50_ = 642 nM) and TRPV1 antagonism (588 nM) compared to COX-2 inhibition (IC_50_ = 18.5 µM), this might mainly drive the *in vivo* anti–inflammatory effects reported for LASSBio-1135.

The signaling pathways involved in TNF-α production by immune cells involves the activation of MAPK cascade, especially p38 MAPK [29]; [37]. The activation of p38 MAPK in both immune cells and in neuronal cells is implicated in most of the modulatories actions of TNF-α during inflammation and pain [38]; [39]. It has been demonstrated that activation of p38 MAPK is related to the TNF-α-mediated bone destruction during arthritis in animal models and the treatment with p38 MAPK inhibitors is able to reduce TNF-α production controlling the inflammatory response [40]; [41]. Thus, LASSBio-1135 inhibited TNF-α production by interfering in the activation of p38 MAPK either inhibiting directly its activation or interfering in the upstream signalling pathway, which means that this compound probably interferes in intracellular signalling to reduce cytokine production. This hypothesis is supported by the scaffold present in LASSBio-1135 molecular structure. The imidazo-pyridine group is present in some inhibitors of protein kinases involved in MAPK signalling pathways, due to its affinity to bind in their ATP pocket [18]. The modulation of p38 MAPK signalling pathway could also control the expression of inflammatory mediators other than TNF-α, such as IL-1β and COX-2, which were not evaluated here and contribute to inflammatory and nociceptive response.

### Multi-targeted Therapy to Manager Chronic Pain

Current therapies to treat patients with chronic pain, such as arthritis-associated pain and several forms of neuropathic pain, consist in using single therapies or some combined therapies which are mostly unsuccessful. Trying to surpass this obstacle, new approaches have been tested to alleviate pain using drug combination or even using multi-target compounds. For instance, the combination of celecoxib, a COX-2 inhibitor, and pregabalin, a calcium-channel blocker, is more efficacious than the monotherapy with either compound alone to treat chronic low-back pain [42]. Similarly, Tributino *et al*. [26] showed that the multi-target compound LASSBio-881, a TRPV1 antagonist which is also CB-ligand and COX inhibitor, is effective in models of chronic pain. LASSBio-1135 may control inflammatory and neuropathic pain through the *in vivo* modulation of immune response and neuronal activity, demonstrated by its effectiveness in reducing inflammatory pain induced by carrageenan dampening TNF-α production and neutrophil migration as well as reducing hyperalgesia induced by nerve injury.

It is not completely clear how TRPV1 activation modulates the inflammatory response, one of the hypotheses resides on the fact that TRPV1 activation drives neurogenic inflammation. In consonance with that, it was shown reduced leukocyte recruitment and bone damage in animal model of arthritis using TRPV1-knockout mice. Conversely animals submitted to inflammatory challenge and treated with TRPV1 agonist reduced the production of cytokines [7]; [43]. However this direct TRPV1 effect in inflammatory response is not enough to explain LASSBio-1135 effect in inflammatory pain model, since we described that this compound also inhibited directly TNF-α in macrophage culture, cells that do not express TRPV1 and strongly drive inflammatory response, with a similar potency as that to antagonize TRPV1 (TNF-α IC_50_ = 642 nM, TRPV1 IC_50_ = 580 nM) [5]; [43]. Thus, LASSBio-1135 likely hampered carrageenan-induced hyperalgesia through both mechanisms: reduction of TNF-α production in immune cells and reduction of neuronal activity by antagonizing TRPV1, mainly at higher doses. This effect is dose-dependent, because the higher efficacy was observed only at 100 µmol.Kg^−1^, even 1 h after treatment, however at 10 µmol.Kg^−1^ was only observed a late effect, which could suggest that at this dose does not occur modulation of both systems.

Similar to inflammatory pain, neuropathic pain also requires immune activation for its development and maintenance. Thus, therapies which control only neuronal activity are not successful in modifying the disease, alleviating only the symptoms (for review [33]). LASSBio-1135 can control targets involved in neuronal and immune activities with the same potency, enhancing its potential applicability in the treatment of neuropathic pain. Even though the treatment was not effective at the first day of treatment, continuous treatment showed to be efficacious, which might reflect either erratic bioavailability or that the effect depends mostly on TNF-α reduction which would take longer to modify the disease. Overall, we suggest that the long-term treatment with LASSBio-1135 during peripheral nerve injury alleviates hyperalgesia by controlling neuronal excitability while modifying the disease by hampering immune activation.

### LASSBio-1135 and the Risks of Being a Multi-targeted Compound

One concern regarding the therapeutic use of multi-targeted compound is the risks of side effects due to their promiscuity. Considering this issue, we investigated the main possible side effects due to the treatment with LASSBio-1135. Clinical trials with TRPV1 antagonists proved their efficacy as analgesics, however they have pointed out hyperthermia as the main undesirable effect of this compounds class [44]. Fortunately, the long-term treatment with LASSBio-1135 did not promote any change in body temperature of mice (Figure 5). This effect could be explained by its multi-targeted characteristic, since at the dose of 100 µmol.Kg^−1^ LASSBio-1135 could counterbalance the hyperthermic effect of the TRPV1 blockage by inhibiting COX-2 (IC_50_ = 18.5 µM), which is known to be involved in regulation of body temperature [2]. We also evaluated if LASSBio-1135 interferes in thermal sensitivity in uninjured mice which did not happen (unpublished data). During long-term treatment with LASSBio-1135 we did not observe change in body weight or behaviour, such as hypolocomotion. Hence, the lack of the commonly observed major side effects for TRPV1 antagonists makes LASSBio-1135 an attractive lead compound with therapeutic potential.

In conclusion, we described here the efficacy of a new multi-targeted compound in models of inflammatory and neuropathic pain. We suggest that the inhibition of targets such as TNF-α, TRPV1 and COX-2 at similar magnitude order contributes to the high efficacy of LASSBio-1135 as well as for reducing the side effects related to interferences in these targets. Our findings suggest that LASSBio-1135 may be a multi-targeted drug prototype to treat chronic pain.

## Supporting Information

Figure S1 **Effect of consecutives applications of CAP in TRPV1-expressing oocytes.** Representative recordings of three subsequent applications of CAP at 1 µM.
(TIF)
