# Supplementary figures and images for: LASSBio-1135: A Dual TRPV1 Antagonist and Anti-TNF-Alpha Compound Orally Effective in Models of Inflammatory and Neuropathic Pain
Source: PLoS One. 2014 Jun 18;9(6):e99510. doi: 10.1371/journal.pone.0099510 (PMC4062389; doi:10.1371/journal.pone.0099510)

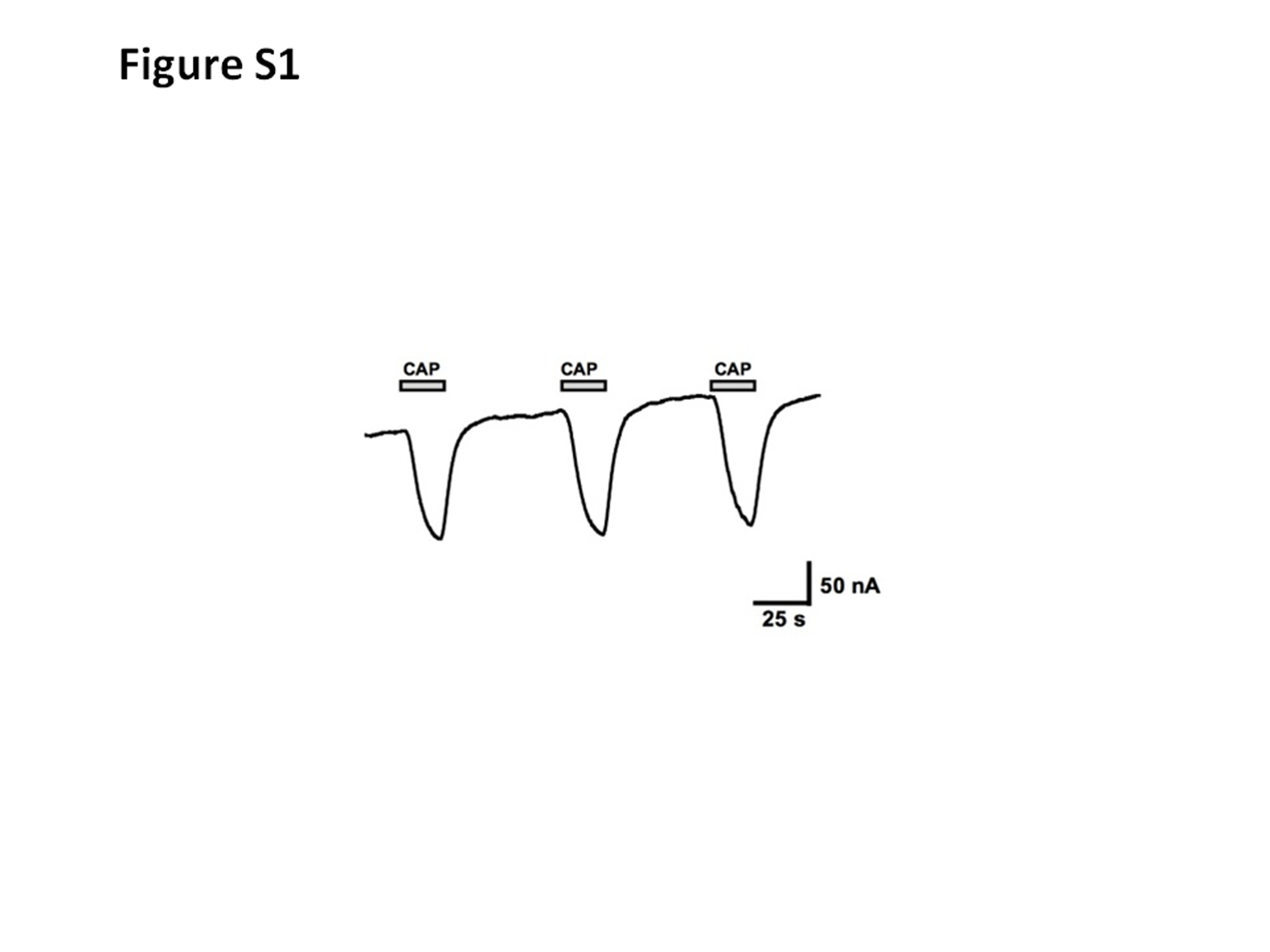

Supplement: Figure S1 — Effect of consecutives applications of CAP in TRPV1-expressing oocytes. Representative recordings of three subsequent applications of CAP at 1 µM. (TIF) [file pone.0099510.s001.tif]
